# The Impact of Early-Stage Chronic Kidney Disease on Weight Loss Outcomes After Gastric Bypass

**DOI:** 10.1007/s11695-023-06862-2

**Published:** 2023-10-10

**Authors:** João Pereira, Pedro R. Pereira, Sara Andrade, Sofia S. Pereira, Mário Nora, Marta Guimarães, Mariana P. Monteiro

**Affiliations:** 1 Unit for Multidisciplinary Research in Biomedicine (UMIB), School of Medicine and Biomedical Sciences (ICBAS), University of Porto, Rua Jorge Viterbo Ferreira 228, 4050-313 Porto, Portugal; 2 ITR-Laboratory of Integrative and Translocation Research in Population Health, Rua das Taipas 135, 4050-600 Porto, Portugal; 3 Department of Nephrology, Hospital de Braga, Rua das Comunidades Lusíadas 133, 4710-243 Braga, Portugal; 4 Department of General Surgery, Hospital São Sebastião, Centro Hospitalar de Entre o Douro e Vouga, Rua Dr. Cândido Pinho, 4050-220 Santa Maria da Feira, Portugal

**Keywords:** Bariatric surgery, Obesity, Focal segmental glomerulosclerosis, Proteinuria, Weight loss, Kidney function tests

## Abstract

**Purpose:**

Weight loss achieved through bariatric metabolic surgery was demonstrated to be effective at reversing chronic kidney dysfunction associated with obesity-related glomerulopathy. However, robust data on how pre-operative kidney status impacts on bariatric metabolic surgery weight loss outcomes is still lacking. The aim of this study was to evaluate the impact of kidney dysfunction on weight loss outcomes after bariatric metabolic surgery.

**Methods:**

Patients with obesity to be submitted to gastric bypass surgery underwent a pre-operative evaluation of creatinine clearance, estimated glomerular filtration rate (eGFR), proteinuria, and albuminuria in 24-hour urine. Body mass index (BMI), % total weight loss (%TWL), and % excess BMI loss (%EBMIL) were assessed at 6 and 12 months after surgery.

**Results:**

Before surgery, patients (*N*=127) had a mean BMI of 39.6 ± 3.0 kg/m^2^, and 56.7% (*n*=72) had a creatinine clearance > 130 mL/min, 23.6% (*n*= 30) presented proteinuria > 150 mg/24h, and 15.0% (*n*= 19) presented albuminuria > 30 mg/24h. After surgery, the mean BMI was 27.7 kg/m^2^ and 25.0 kg/m^2^ at 6 and 12 months, respectively (*p*<0.0001). The %TWL was lower in patients with pre-operative eGFR < percentile 25 (34.4 ± 5.8% vs 39.4 ± 4.9%, *p*=0.0007, at 12 months). There were no significant correlations between weight loss metrics and pre-operative creatinine clearance rate, proteinuria, or albuminuria.

**Conclusion:**

Early-stage chronic kidney disease (G2) has a negative impact on short-term weight loss outcomes after bariatric metabolic surgery, albeit in a magnitude inferior to the clinically relevant threshold.

**Graphical abstract:**

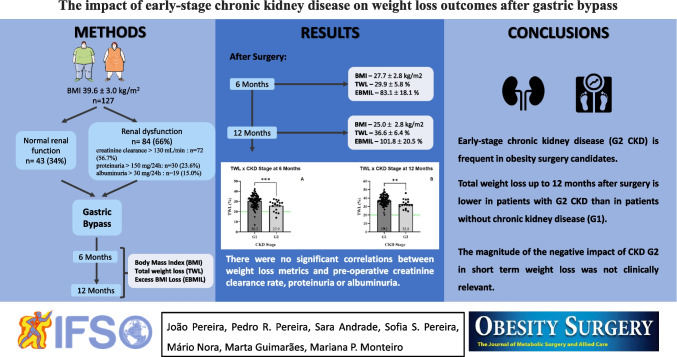

**Supplementary Information:**

The online version contains supplementary material available at 10.1007/s11695-023-06862-2.

## Introduction

The weight loss and obesity-associated medical problems improvements achieved through bariatric metabolic surgery are known to be influenced both by pre-operative and post-operative patient characteristics [1]. The pre-operative factors recognized to have the greatest impact on weight loss are pre-operative body weight and body mass index (BMI), presence of eating disorders, such as binge eating or food addiction [2, 3], presence of type 2 diabetes mellitus [3], residual β-cell function [4], glycated hemoglobin (HbA1c), and fasting blood glucose levels [1]; additionally, genetic, dietary, and anatomic factors were also shown to have a potential impact on bariatric metabolic surgery weight loss outcomes, although the evidence is less robust [3, 5].

Obesity is associated with a form of glomerular involvement coined obesity-related glomerulopathy [4]. In the absence of histological confirmation, slowly progressive proteinuria within the subnephrotic range in a patient with obesity can be used as a surrogate diagnostic marker for ORG, provided other causes of renal disease are not suspected [6]. ORG typical features include glomerulomegaly and focal segmental glomerulosclerosis, which is predominantly perihilar, although it can affect any portion of the glomerulus [7]. Studies suggest that the perihilar sclerosis predominance could be explained by the increased filtration demands and dilation of the afferent arteriole, which leads to an increase in ultrafiltration pressure [8]. As disease progresses, glomeruli enlarge and podocytes’ density decreases, aggravating the segmental sclerosis and the overall renal function [9–11]. Although most patients have stable or slowly progressive proteinuria, up to one-third of individuals can develop progressive renal failure and end-stage renal disease (ESRD) [7, 12–14]. Notwithstanding, studies have shown that hyperfiltration and proteinuria can be improved, provided sustained weight loss is achieved either with medical interventions or with bariatric metabolic surgery [6].

Patients with advanced chronic kidney disease (CKD) (stages 4 and 5) have been shown to experience less weight loss, with worse weight loss outcomes at 6 and 12 months after bariatric metabolic surgery, when compared to patients in less-severe CKD stages [15]. Although the mechanisms for this finding are still lacking, factors such as the impact of malnutrition and the obesity paradox in CKD [16], decreased energy expenditure in advanced CKD [17], and the impending need for a kidney transplant have been appointed as possibly implicated [15]. In contrast, in another study, no significant differences at 6 and 12 months after surgery were observed for patients in CKD stages 1, 2, 3a, and 3b [18]. However, previous studies were retrospective and used CKD-EPI equations indexed to body surface area to estimate glomerular filtration rate (GFR), as a surrogate measure of kidney function, which is less reliable in patients with obesity [6, 19]. Additionally, none of the previous studies evaluated albuminuria or proteinuria, and both included patients with type 2 diabetes mellitus, features that could have impacted on data interpretation.

In order to contribute for filling this knowledge gap, the aim of this study was to evaluate the impact of pre-operative kidney function on weight loss outcomes after bariatric metabolic surgery.

## Methods

### Study Population

Patients enrolled in this study attended a non-academic hospital among obesity surgery candidates from 2019 to 2022. Patients with obesity were considered eligible for bariatric metabolic surgery if presented a BMI higher than 40 kg/m^2^ or a BMI higher than 35 kg/m^2^ in the presence of obesity-associated medical problems, provided there were no contraindications for the procedure. Bariatric metabolic surgery candidates who accepted to participate underwent a 24-hour urine analysis to assess creatinine, albumin, and total proteins, in addition to the pre-established pre-operative routine evaluations, which included complete blood count, fasting blood glucose, HbA1c, lipid profile, creatinine, urea, albumin, and total protein serum levels.

Patient candidates for revision bariatric metabolic surgery procedures or submitted to any bariatric metabolic surgery procedure other than gastric bypass were excluded from the study. Patients with a previous history of gestational diabetes, or current diagnosis of diabetes defined as having an HbA1c ≥ 6.5% or treated with anti-diabetic drugs, regardless of HbA1c levels (*n*=15), were also excluded, since these conditions were previously demonstrated to have a negative impact on bariatric metabolic surgery weight loss outcomes. Moreover, in order to avoid the potential risk of bias derived from inadequate 24-h urine collection, patients with a 24-urine collection volume under 800 mL were also excluded, leading to the final number of patients included in this study for data analysis.

All surgical procedures were done by laparoscopy. Gastric bypass consisted in performing a 15-mL gastric pouch by transecting the lesser curvature of the stomach 5 cm distally to the cardia and transected again vertically in direction of the cardia while avoiding the esophagus. Afterwards, a calibrated gastroileal anastomosis and an ileal–ileal anastomosis were made, thus creating a biliopancreatic limb with a variable length from (50 to 200 cm) and a constant alimentary limb with 120 cm, as previously described in further detail [20].

### Data Collection

Data concerning preoperative body weight, height, BMI, associated medical problems, ongoing medications, blood pressure, complete blood count, fasting blood glucose, HbA1c, lipid profile, creatinine, urea, albumin, and total protein serum levels were collected and registered. The patients were given oral and written instructions on how to perform the 24-hour urine collection.

The 24-hour creatinine clearance [(urine creatinine concentration [mg/dL] × total urine volume [mL/24]) ÷ (serum creatinine concentration [mg/dL] × 1440)], along with the eGFR, calculated with the 2021 CKD-EPI creatinine equation [21], was used to assess kidney function.

Anthropometric data was used to calculate BMI [weight (kg) ÷ height^2^ (m^2^)], percentage of EBMIL [(preoperative BMI − postoperative BMI) ÷ (preoperative BMI − 25) × 100] and percentage of TWL [(preoperative weight − postoperative weight) ÷ preoperative weight × 100].

After bariatric metabolic surgery, patients were followed by a multidisciplinary team which included surgeons, endocrinologists, psychologists, and nutritionists. Patients underwent a revaluation of the aforementioned parameters during routine follow-up visits at 6 and 12 months after surgery. The primary outcomes of this study were % total weight loss (%TWL) and % excess BMI loss (%EBMIL).

### Statistical Analysis

Continuous variables are expressed as mean ± standard deviation (SD) and categorical values as percentage, unless otherwise specified. The Kolmogorov-Smirnov test was used to determine the normality of the continuous variables. Comparison of two independent variables was performed by using either the Pearson correlation coefficient or the Spearman correlation coefficient, as appropriate depending on the normality. A correlation coefficient value of 0–0.19 is assumed as very weak, 0.2–0.39 as weak, 0.40–0.59 as moderate, 0.6–0.79 as strong, and 0.8–1 as very strong correlation. Statistically significant (*p*<0.05) correlations were further investigated with univariate and forward multivariate regression analyses. To compare two non-parametric variables, the Mann-Whitney test was used, while the unpaired *t*-test was used to compare two parametric variables. To compare three or more variables, the one-way ANOVA test or the Kruskal-Wallis test was performed, according to the normality distribution of the variables. A *p*-value < 0.05 was considered statistically significant. Statistical analysis was carried out using GraphPad (Prism; Version 9.5.1) and SPSS (IBM; Version 27.0) for Windows.

The power of the study was estimated to detect a difference of 5% in our primary outcome (total weight loss 12 months after surgery, corresponding to the minimum weight loss threshold to be considered clinically relevant [22], with an alpha value of 0.05. The power of the study was calculated using the PS Power and Sample Size Calculations, for Windows.

## Results

### Patients

**Fig. 1:**
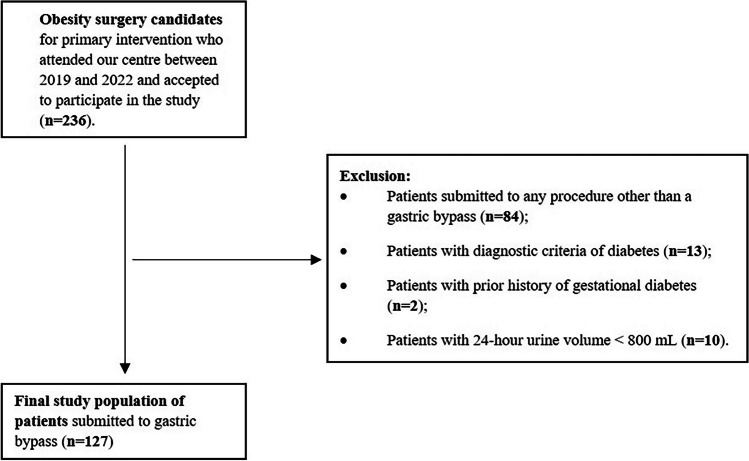
Patients’ selection criteria

Of the initial bariatric metabolic surgery candidates (*n*=236) who accepted to participate in this study, patients were excluded for having been submitted to procedures other than gastric bypass (*n*=84), for having  a previous history of gestational diabetes or established diabetes before surgery (*n*=15) and for having a 24-urine collection volume under 800 mL (*n*=10) (Fig. 1).

Our final patient cohort (*n*=127) encompassed predominantly females (*n*=104; 81.9%) over males (*n*=23; 18.1%), in parallel to the sex proportion of patients reported to attend obesity clinics [23, 24]. There were no follow-up dropouts reported. At the time of surgery, the patients’ mean age was 42.7 ± 10.9 years, and the mean BMI was 39.6 ± 3.0 kg/m^2^. Detailed patients’ clinical and biochemical characteristics are depicted in Table 1.

**Table 1:** Patients’ preoperative demographic, clinical, and analytical characterization

		Total (*n*=127)
Age (years)		42.7 ± 10.9
Sex	Male (*n*, %)	23 (18.1%)
	Female (*n*, %)	104 (81.9%)
Weight (kg)		106.4 ± 13.2
BMI (kg/m^2^)		39.6 ± 3.0
Hypertension	No (*n*, %)	94 (74.0%)
	Yes (*n*, %)	33 (26.0%)
Dyslipidemia	No (*n*, %)	93 (73.2%)
	Yes (*n*, %)	34 (26.8%)
ACE-i	No (*n*, %)	115 (90.6%)
	One (*n*, %)	12 (9.5%)
ARB	No (*n*, %)	111 (87.4%)
	One (*n*, %)	16 (12.6%)
CCB dihydropyridine	No (*n*, %)	118 (92.9%)
	One (*n*, %)	9 (7.1%)
Diuretics	No (*n*, %)	111 (87.4%)
	One (*n*, %)	14 (11.0%)
	Two (*n*, %)	2 (1.6%)
Glucose (mg/dL)		93.5 ± 27.3
Glycated hemoglobin (%)		5.4 ± 0.8
Serum urea (mg/dL)		30.9 ± 7.6
Serum creatinine (mg/dL)		0.8 ± 0.1
Total cholesterol (mg/dL)		195.5 ± 34.7
HDL-c (mg/dL)		50.0 ± 11.6
LDL-c (mg/dL)		132.9 ± 35.4
VLDL-c (mg/dL)		26.2 ± 12.8
Triglycerides (mg/dL)		130.9 ± 64.4
Total proteins (mg/dL)		7.0 ± 0.3
24-h diuresis (mL)		1566.0 ± 534.8
24-h albuminuria (mg/24h)		21.3 ± 32.0
24-h proteinuria (mg/24h)		120.2 ± 66.2
24-h urine creatinine (mg/24h)		1582.2 ± 509.7
eGFR (2021 CKD-EPI creatinine [18])		105.0 ± 12.9
Creatinine clearance (mL/min)		145.3 ± 39.2
Creatinine clearance category	< 90 mL/min, *n* (%)	4 (3.2%)
	90–130 mL/min, *n* (%)	51 (40.2%)
	130–170 mL/min, *n* (%)	37 (29.1%)
	> 170 mL/min, *n* (%)	35 (27.6%)
24-h proteinuria category	<150 mg/24h, *n* (%)	97 (76.4%)
	>150 mg/24h, *n* (%)	30 (23.6%)
24-h albuminuria category	<30 mg/24h, *n* (%)	108 (85.0%)
	30–300 mg/24h, *n* (%)	19 (15.0%)
	>300 mg/24h, *n* (%)	0 (0.0%)

Continuous variables are presented as mean ± standard deviation. Nominal variables are presented in number (percentage)

*BMI* body mass index, *ACE-i* angiotensin conversing enzyme inhibitors, *ARB* angiotensin II receptor blockers, *CCB* calcium channel blockers, *HDL-c* high density lipoprotein, *LDL-c* low density lipoprotein, *VLDL-c* very-low density lipoprotein, *eGFR* estimated glomerular filtration rate, *CKD-EPI* chronic kidney disease-epidemiology

### Postoperative Anthropometric Characteristics

After bariatric metabolic surgery, there was a statistically significant reduction in body weight and BMI (39.6 kg/m^2^ pre-operatively, 27.7 kg/m^2^ at 6 months, and 25.0 kg/m^2^ at 12 months, *p*<0.0001), yielding a %TWL of 29.9 % and 36.6 % and %EBMIL of 83.1 % and 101.8 % at 6 and 12 months after surgery, respectively. Additionally, only 3 patients (2.4%) did not achieve a %EBMIL greater than 50% at 6 months and only 1 patient (0.8%) at 12 months after surgery (Table 2).

**Table 2:** Patients’ anthropometric parameters before and 6 months and 12 months after bariatric surgery

		Pre-Op	6 months	12 months	*p*-value
Weight (kg)		106.4 ± 13.2	74.5 ± 9.2	66.9 ± 9.7	**<0.0001**
BMI (kg/m^2^)		39.6 ± 3.0	27.7 ± 2.8	25.0 ± 2.8	**<0.0001**
%TWL		–	29.9 ± 5.8	36.6 ± 6.4	**<0.0001**
%EBMIL		–	83.1 ± 18.1	101.8 ± 20.5	**<0.0001**
%EBMIL failure/success criteria	Missing Values	–	2 (1.6%)	9 (7.1%)	–
	EBMIL < 50 % Failure	–	3 (2.4%)	1 (0.8%)	–
	EBMIL > 50 Success	–	122 (96.1%)	117 (92.1%)	–

Variables are presented as mean ± standard deviation. *p*-values refer to all possible comparisons between the three different follow-up stages. Significant differences are highlighted in bold. The “missing values” of EBMIL success criteria refer to patients who skipped the corresponding clinical evaluation

*BMI* body mass index, %*TWL* % total weight loss, *%EBMIL* % excess BMI loss

### Relationship Between Kidney Function Markers and Weight Loss Parameters

There were no statistically significant differences in %TWL and %EBMIL at any time point of the follow-up, when comparing patients with different creatinine clearance categories (Table 3). The same was observed when comparing patients with greater creatinine clearance rates (higher than percentile 75 [>172.6 mL/min]), with those with lower creatinine clearance rates (lesser than percentile 25 [<113.9 mL/min]) (supplementary table 1).

**Table 3:** Weight Loss according to pre-operative creatinine clearance’s categories

	Creatinine clearance category				*p*-value
	< 90 mL/min	90–130 mL/min	130–170 mL/min	> 170 mL/min	
%TWL 6 months	24.1 ± 6.4	29.4 ± 5.1	30.1 ± 5.9	31.0 ± 5.4	> 0.05
%EBMIL 6 months	65.3 ± 32.4	83.9 ± 16.2	81.8 ± 17.9	85.3 ± 18.5	> 0.05
%TWL 12 months	29.6 ± 17.9	36.4 ± 5.3	36.1 ± 6.4	38.3 ± 5.2	> 0.05
%EBMIL 12 months	80.2 ± 46.7	103.3 ± 18.3	98.4 ± 17.0	106.0 ± 21.8	> 0.05

Variables are presented as mean ± standard deviation. *p*-values refer to all possible comparisons between the four different categories

*%TWL* % total weight loss, *%EBMIL* % excess BMI loss

**Fig. 2:**
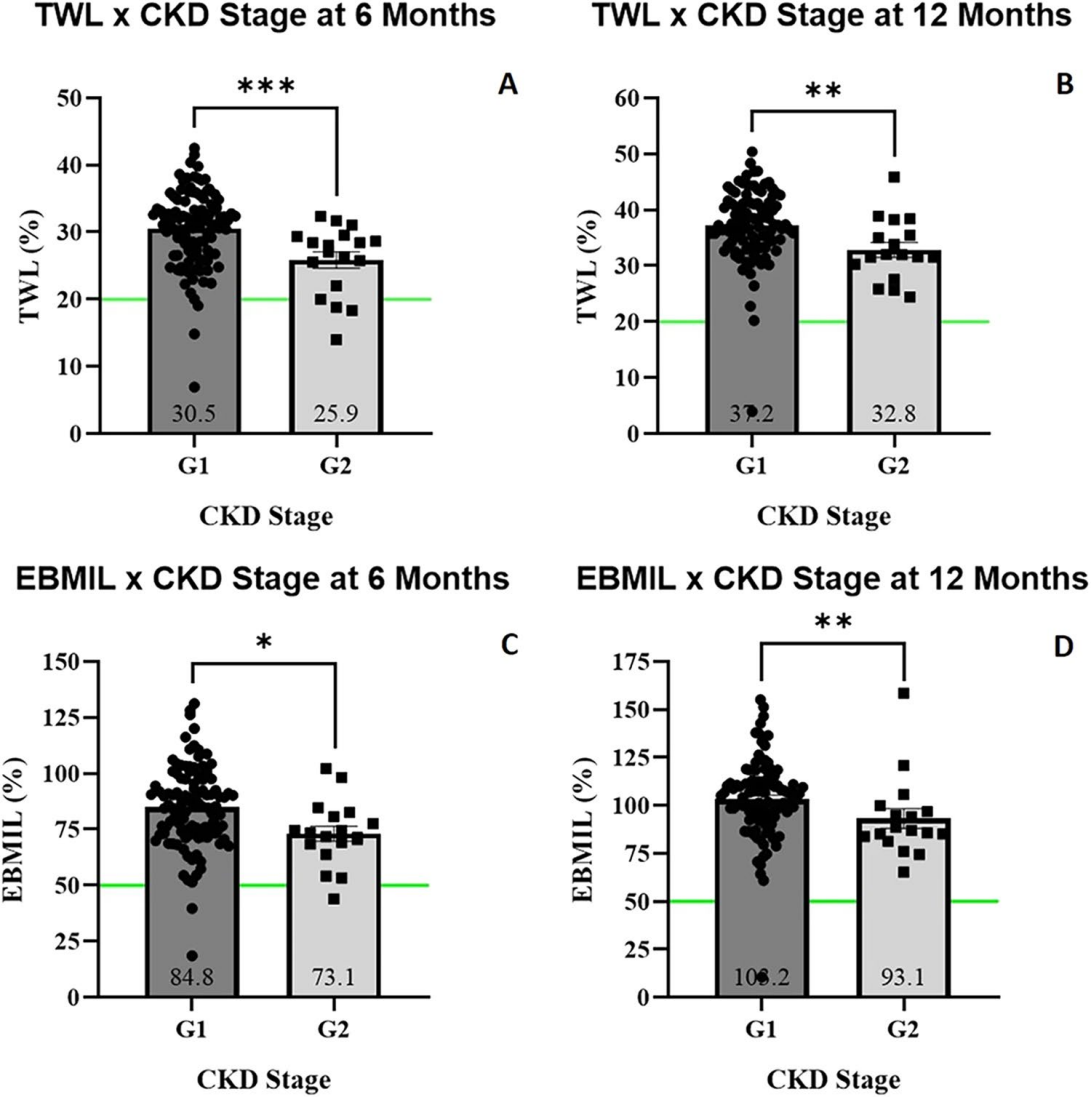
Anthropometric evolution according to CKD stage. %TWL 6 months after surgery (**A**); %TWL 12 months after surgery (**B**); %EBMIL 6 months after surgery (**C**); %EBMIL 12 months after surgery (**D**). %TWL, % total weight loss; %EBMIL, % excess BMI loss; CKD, chronic kidney disease

Patients with pre-operative stage G2 CKD had lower %TWL (25.9 ± 5.2% *versus* 30.5 ± 5.7%, *p*=0.0006, at 6 months; 32.8 ± 5.5% *versus* 37.2 ± 6.3%, *p*=0.0016, at 12 months), and %EBMIL (73.1 ± 14.4% *versus* 84.8 ± 18.1%, *p*=0.0104, at 6 months; 93.1 ± 21.1% *versus* 103.2 ± 20.2%, *p*=0.0037, at 12 months), when compared to patients without pre-operative CKD (stage G1) (Table 4; Fig. 2). Patients with lower eGFR values (lesser than percentile 25 [<98.3 mL/min]) also had lower %TWL (27.6 ± 5.7% *versus* 31.8 ± 5.1%, *p*=0.0031, at 6 months; 34.4 ± 5.8% *versus* 39.4 ± 4.9%, *p*=0.0007, at 12 months) and %EBMIL (76.3 ± 17.5% *versus* 85.1 ± 17.5%, *p*=0.0478, at 6 months; 94.8 ± 19.8% *versus* 106.1 ± 19.1%, *p*=0.0061, at 12 months), when compared to patients with greater eGFR values (higher than percentile 75 [>114.2 mL/min]) (supplementary table 2; supplementary figure 1).

**Table 4:** Weight loss according to pre-operative CKD stages (according to eGFR)

	CKD stage		*p*-value
	**G1**	**G2**	
%TWL 6 months	30.5 ± 5.7	25.9 ± 5.2	**0.0006**
%EBMIL 6 months	84.8 ± 18.1	73.1 ± 14.4	**0.0104**
%TWL 12 months	37.2 ± 6.3	32.8 ± 5.5	**0.0016**
%EBMIL 12 months	103.2 ± 20.2	93.1 ± 21.1	**0.0037**

Variables are presented as mean ± standard deviation. Significant differences are highlighted in bold

*CKD* chronic kidney disease, *%TWL* % total weight loss, *%EBMIL* % excess BMI loss

A weak positive correlation between pre-operative eGFR and %TWL at 6 months (*r*=0.238, *r*^2^=5.7%, *p*=0.008) and at 12 months (*r*=0.261, *r*^2^=6.8%, *p*=0.004), as well as with %EBMIL at 12 months after surgery (*r*=0.244, *r*^2^=5.9%, *p*=0.008) was found. Very weak and weak positive correlations between pre-operative creatinine clearance rate and %TWL at 6 months (*r*=0.189, *r*^2^=3.6%, *p*=0.035) and at 12 months (*r*=0.201, *r*^2^=4.0%, *p*=0.029), respectively, were also observed. No statistically significant correlations between pre-operative creatinine clearance rate and %EBMIL were found, neither at 6 nor 12 months after surgery. No statistically significant correlations were found between pre-operative 24h proteinuria or albuminuria and the %TWL and %EBMIL at 6 and 12 months after surgery (Tables 5 and 6).

**Table 5:** Weight loss according to pre-operative albuminuria’s and proteinuria’s classification

	Albuminuria classification			Proteinuria classification		
	< 30 mg/24h	> 30 mg/24h	*p*-value	< 150 mg/24h	> 150 mg/24h	*p*-value
%TWL 6 months	29.9 ± 5.3	29.7 ± 8.5	0.6820	30.0 ± 5.3	29.3 ± 7.3	0.6721
%EBMIL 6 months	82.7 ± 17.1	85.0 ± 23.6	0.3472	82.4 ± 16.5	85.3 ± 22.8	0.5430
%TWL 12 months	40.0 ± 6.5	34.4 ± 5.4	0.0712	37.1 ± 6.5	35.3 ± 5.5	0.0702
%EBMIL 12 months	102.3 ± 21.1	98.8 ± 16.9	0.7314	101.8 ± 21.5	101.6 ± 17.4	0.7140

Variables are presented as mean ± standard deviation

*%TWL* % total weight loss, *%EBMIL* % excess BMI loss

**Table 6:** Correlations between weight loss metrics and kidney function markers

	2021 CKD-EPI creatinine-eGFR	Creatinine clearance	Proteinuria	Albuminuria
%TWL 6 months	*R*=0.238 **(*p*=0.008)**	*R*=0.189 **(*p*=0.035)**	*R*=−0.029 (*p*=0.749)	*R*=0.136 (*p*=0.129)
%EBMIL 6 months	*R*=0.171 (*p*=0.057)	*R*=0.063 (*p*=0.482)	*R*=0.012 (*p*=0.898)	*R*=0.126 (*p*=0.161)
%TWL 12 months	*R*=0.261 (***p*=0.004**)	*R*=0.201 (***p*=0.029**)	*R*=−0.117 (*p*=0.208)	*R*=−0.084 (*p*=0.364)
%EBMIL 12 months	*R*=0.244 (***p*=0.008**)	*R*=0.062 (*p*=0.508)	*R*=0.025 (*p*=0.786)	*R*=−0.006 (*p*=0.950)

“*R*” correlation’s coefficient, Pearson, or Spearman, as applicable. Significant differences are highlighted in bold

*%TWL* % total weight loss, *%EBMIL* % excess BMI loss, *CKD-EPI* chronic kidney disease-epidemiology, *eGFR* estimated glomerular filtration rate

Univariate linear regression models were used to further evaluate the statistically significant correlations. With reference to eGFR and %TWL at 6 months, the fitted regression model was as follows: %TWL = 18.693 + 0.106 × eGFR, *p* = 0.008. Regarding eGFR and %TWL at 12 months, the fitted regression model was as follows: %TWL = 23.023 + 0.130 × eGFR, *p* = 0.004. Concerning eGFR and %EBMIL at 12 months, the fitted regression model was as follows: %EBMIL = 69.085 + 0.313 × eGFR, *p* = 0.034. As for creatinine clearance and %TWL at 6 months, the fitted regression model was as follows: %TWL = 25.931 + 0.027 × creatinine clearance, *p* = 0.041. For creatinine clearance and %TWL at 12 months, the fitted regression model was as follows: %TWL = 32.347 + 0.029 × creatinine clearance, *p* = 0.051.

Additionally, multivariate regression analyses found that eGFR predicted %TWL at 12 months (*β* = 0.296, *p* = 0.008). No other statistically significant associations were found when performing multivariate regression analyses between the other aforementioned renal function markers and weight loss metrics.

## Discussion

The weight loss efficacy of bariatric metabolic surgery is known to be influenced by a variety of pre-operative factors. Several patient characteristics were identified as being determinants of weight loss and obesity-associated medical problem remission after bariatric metabolic surgery, among these diabetes is one of the most impactful [1, 3]. Additionally, previous reports also suggest that patients with an eGFR under 30 mL/min/1.73m^2^ tend to have worse weight loss outcomes after bariatric surgery [15, 18]. However, the available evidence is derived from studies that presented some limitations, namely, the concomitant presence of dysglycemia, which could have impacted on data interpretation [1, 3]. The aim of this study was to investigate whether bariatric metabolic surgery weight loss is influenced by creatinine clearance rate, eGFR, proteinuria, or albuminuria, in patients without the concomitant presence of dysglycemia.

Previous studies suggested that patients with severe CKD experience a lower weight loss efficacy after bariatric metabolic surgery [15], which has been attributed to several factors. One of these factors is the need for urgent weight loss in the setting of a potential kidney transplant surgery in patients with advanced CKD, which might account for worst preoperative management of care, thus hampering surgery outcomes. Another factor is the high prevalence of malnutrition, which is a major complication of advanced CKD [25] and can influence dietary choices and weight loss objectives for this patient population. Also, the obesity paradox in CKD, which proposes obesity protects against adverse health outcomes in the setting of advanced CKD, might influence the physician perspective on weight loss targets for this particular patient population [26]. Additionally, reduced resting and physical activity energy expenditure in patients with advanced CKD, as a consequence of patient frailty, sedentary lifestyle, reduced lean body mass, and advanced-CKD-associated sarcopenia, are also potentially contributing factors. In order to evaluate the impact of pre-operative kidney function on weight loss outcomes after bariatric metabolic surgery, we assessed whether the abovementioned markers of kidney dysfunction had a significant impact on weight loss outcomes after bariatric metabolic surgery. Our data showed that there were no significant differences in weight loss metrics when patients with and without glomerular hyperfiltration were compared, as well as there were no significant correlations between pre-operative creatinine clearance and any of the weight-loss metrics evaluated. This being said, it seems that in patients with obesity and without concomitant diabetes, pre-operative creatinine clearance and hyperfiltration do not seem to have any clinically relevant effect on weight loss outcomes after gastric bypass surgery, at least up to 12 months after the procedure. Nevertheless, this observation does not preclude that no differences will be observed over time, for which mid and long-term studies are still needed. Of notice, is the fact that patients with lower eGFR values (lesser than percentile 25 [<98.3 mL/min]) also had lower %TWL (34.4 ± 5.8% *vs* 39.4 ± 4.9%, at 12 months) and %EBMIL (94.8 ± 19.8% *vs* 106.1 ± 19.1%, at 12 months), when compared to patients with greater eGFR values (higher than percentile 75 [>114.2 mL/min]). Notwithstanding the weight loss differences observed, with the sole exception of a patient without any pre-operative abnormality in kidney function markers, all patients fulfilled the Halverson and Koehler 50% EBMIL criteria to define successful weight loss after bariatric interventions [27]. Moreover, no strong correlations were identified between eGFR and %TWL or %EBMIL at 6 and 12 months after bariatric metabolic surgery. Additionally, no clinically significant associations were observed in univariate and multivariate regression analyses regarding creatinine clearance, eGFR and weight loss metrics. Overall, these findings suggest that regardless the statically significant differences in weight loss achieved by patients with lower eGFR and CKD stage 2, this negative impact on weight-loss achieved up to 12 months after bariatric metabolic surgery is under the 5 % threshold to be considered clinically relevant [28], therefore corroborating previously available evidence [18]. Furthermore, our data did not show any significant weight loss differences when comparing patients with different albuminuria and proteinuria categories, nor significant correlations between preoperative proteinuria and albuminuria and weight loss metrics. These findings suggest that even patients with clinically significant proteinuria (greater than 150 mg/24h) or albuminuria (greater than 30 mg/24h), which are frequent findings in ORG [12], benefit from the weight loss achieved with gastric bypass surgery, besides the improvements in kidney function documented in previous studies[29–36].

Over half of our patient population (56.7%, *n*=72) presented a pre-operative creatinine clearance greater than 130 mL/min. Even though there is no universally accepted threshold to define glomerular hyperfiltration, several studies advocate that this threshold should fall within the interval from 125 to 175 mL/min/1.73m^2^ [37]. Therefore, if this creatinine clearance rate interval is considered, approximately half of our bariatric metabolic surgery candidates are likely to have glomerular hyperfiltration, which renders a particular risk for kidney injury, even in the absence of diabetes [6]. Glomerular hyperfiltration plays a central role in the development and progression of ORG [6]. Patients with obesity tend to depict renin-angiotensin-aldosterone system (RAAS) overactivation [38], in result of the increased production of its substrates by adipocytes [39, 40], as well as from renal sympathetic nervous system overactivation [41]. In turn, RAAS activation leads to excessive tubular reabsorption of sodium and water [42, 43] that is positively correlated with BMI and waist circumference [44], decreased distal solute delivery, activation of tubuloglomerular feedback and vasodilation of the afferent arteriole, which in turn lead to increased transcapillary hydraulic pressure gradient with consequent glomerular hyperfiltration [45].

Additionally, proteinuria over 150 mg/24h and albuminuria over 30 mg/24h were identified in 23.4% and 15.0% of our study patients, respectively. The prevalence of proteinuria and albuminuria observed in our patient population is consistent with previous reports [46]. Proteinuria and albuminuria are believed to be a long term consequence of the increased transcapillary hydraulic pressure and glomerular hyperfiltration, which induces glomerulomegaly through an increase in circumferential and axial capillary wall stress, glomerular tuft enlargement and expansion of the glomerular basal membrane [47]. In turn, this causes a fluid shear stress on podocytes, prompting maladaptive hypertrophy and leading to podocyte effacement, detachment, and apoptosis with consequent glomerulosclerosis [47]. Glomerular structural changes [48], combined with the low circulating adiponectin [49] and the glomerular permselectivity effect of angiotensin II [50], were appointed as contributing factors for the increased prevalence of albuminuria and subnephrotic proteinuria in patients with obesity [51]. Therefore, our data clearly shows that patients with obesity, without the confounding effect of diabetes, depict not only signs of glomerular hyperfiltration, but also surrogate signs of altered renal structure and physiology. The herein study data corroborates our previous report in a larger cohort that a considerable proportion of patients with obesity undergoing bariatric interventions present unsuspected signs of kidney function deterioration [24]. The prevalence of kidney dysfunction identified in these patients reinforces the need for considering including a 24-hour urine analysis on routine pre-operative workout. This simple measure would allow a comprehensive assessment of obesity-associated medical problems to categorize patient risk, enable a timely diagnosis of kidney dysfunction, and ultimately contribute towards improving patient care by monitoring renal function markers, adopting renoprotective measures [7, 12] and reducing proteinuria [6].

This study presents some limitations that must be acknowledged, since these could have impacted on our data interpretation. The eGFR was calculated with the 2021 CKD-EPI creatinine equation, which is indexed for body surface area (BSA) and therefore is not the best marker to estimate kidney function and GFR in a population of patients with obesity, as pointed by previous studies [6, 19]. Additionally, since our sample size was rather limited, although sufficiently large to disclose some robust statistical differences, these findings require confirmatory studies in larger patient populations. Nevertheless, for all study’s variables (albuminuria, proteinuria, CKD-EPI 2021), with the exception of creatinine clearance, the study’s power was higher than 85% to detect a difference higher than 5% in TWL 12 months after surgery, between the respective categories. Nonetheless, our study design also holds some strengths that bolster its accuracy on the proposed objectives, when compared to previously available data [15, 18]. In order to prevent underestimation of GFR in a population of patients with obesity which would conceal glomerular hyperfiltration, creatinine clearance calculation was not corrected for BSA [19]. Therefore, we are confident that our findings reveal a more accurate estimate of glomerular filtration rate for this patient population. In addition, we evaluated proteinuria and albuminuria through 24-hour urine collection, increasing our ability to analyze small variations in albumin and protein excretion rates, as opposed to the alternative use of spot urine analysis, and we also excluded patients with concomitant diabetes, which could have impacted on our data interpretation.

## Conclusion

In summary, this study demonstrates that the presence of hyperfiltration, proteinuria or albuminuria, does not seem to have a clinically relevant negative impact on short-term weight loss outcomes after bariatric metabolic surgery. These findings, if confirmed over longer period, suggest that patients with obesity and concomitant early-stage (G2) deterioration of kidney function can still achieve satisfactory weight loss outcomes after bariatric metabolic surgery, while potentially benefiting from renal function improvements as previously reported.

### Supplementary Information

ESM 1: Figure S1. Anthropometric evolution according to eGFR percentiles (P25: 98.3 mL/min; P75: 114.2 mL/min). %TWL 6 months after surgery (**A**); %TWL 12 months after surgery (**B**); %EBMIL 6 months after surgery (**C**); %EBMIL 12 months after surgery (**D**). Abbreviations: %TWL, % total weight loss; %EBMIL, % excess BMI loss; eGFR, estimated glomerular filtration rate; CKD-EPI, chronic kidney disease - epidemiology. (TIF 3455 kb)

ESM 2: Table S1. Weight loss according to pre-operative creatinine clearance extreme values (DOCX 14 kb)

ESM 3: Table S2. Weight loss according to pre-operative eGFR percentile (DOCX 14 kb)

## Data Availability

The datasets generated during and/or analysed during the current study are available from the corresponding author on reasonable request.
