# Supplementary figures and images for: The Impact of Early-Stage Chronic Kidney Disease on Weight Loss Outcomes After Gastric Bypass
Source: Obes Surg. 2023 Oct 10;33(12):3767–77. doi: 10.1007/s11695-023-06862-2 (PMC10687110; doi:10.1007/s11695-023-06862-2)

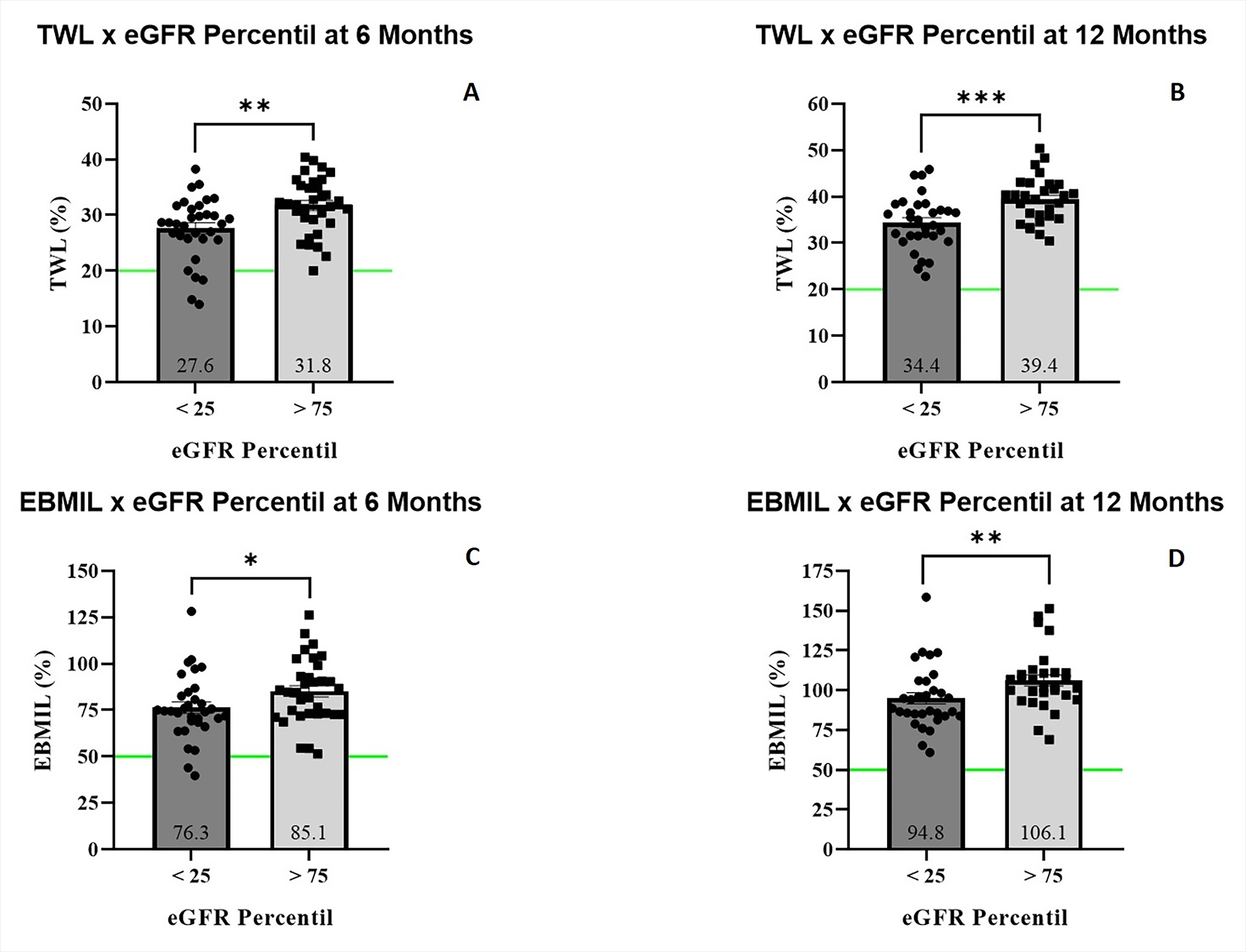

Supplement: Supplementary file 1 — Figure S1. Anthropometric evolution according to eGFR percentiles (P25: 98.3 mL/min; P75: 114.2 mL/min). %TWL 6 months after surgery (A); %TWL 12 months after surgery (B); %EBMIL 6 months after surgery (C); %EBMIL 12 months after surgery (D). Abbreviations: %TWL, % total weight loss; %EBMIL, % excess BMI loss; eGFR, estimated glomerular filtration rate; CKD-EPI, chronic kidney disease - epidemiology. (TIF 3455 kb) [file 11695_2023_6862_MOESM1_ESM.tif]
