# Supplementary material for: The Impact of Early-Stage Chronic Kidney Disease on Weight Loss Outcomes After Gastric Bypass
Source: Obes Surg. 2023 Oct 10;33(12):3767–77. doi: 10.1007/s11695-023-06862-2 (PMC10687110; doi:10.1007/s11695-023-06862-2)
Supplement: Supplementary file 2 — Table S1. Weight loss according to pre-operative creatinine clearance extreme values (DOCX 14 kb) [file 11695_2023_6862_MOESM2_ESM.docx]

**Supplementary Table 1 –** Weight loss according to pre-operative creatinine clearance extreme values.

|  | Creatinine Clearance Percentile | | p-value |
| --- | --- | --- | --- |
|  | **< P25** | **> P75** |  |
| %TWL 6 Months | 28.7 ± 6.2 | 31.0 ± 5.5 | 0.0876 |
| %EBMIL 6 Months | 81.2 ± 19.7 | 85.2 ± 18.9 | 0.4427 |
| %TWL 12 Months | 34.9 ± 8.0 | 38.0 ± 5.2 | 0.0615 |
| %EBMIL 12 Months | 98.5 ± 24.4 | 105.4 ± 22.4 | 0.4102 |

Variables are presented in mean ± standard deviation. P25: 113.9 mL/min; P75: 172.6 mL/min.

Abbreviations: %TWL, % total weight loss; %EBMIL, % excess BMI loss.
