# Supplementary material for: The Impact of Early-Stage Chronic Kidney Disease on Weight Loss Outcomes After Gastric Bypass
Source: Obes Surg. 2023 Oct 10;33(12):3767–77. doi: 10.1007/s11695-023-06862-2 (PMC10687110; doi:10.1007/s11695-023-06862-2)
Supplement: Supplementary file 3 — Table S2. Weight loss according to pre-operative eGFR percentile (DOCX 14 kb) [file 11695_2023_6862_MOESM3_ESM.docx]

**Supplementary Table 2 –** Weight loss according to pre-operative eGFR percentile.

|  | eGFR Percentile | | p-value |
| --- | --- | --- | --- |
|  | **< P25** | **> P75** |  |
| %TWL 6 Months | 27.6 ± 5.7 | 31.8 ± 5.1 | **0.0031** |
| %EBMIL 6 Months | 76.3 ± 17.5 | 85.1 ± 17.5 | **0.0478** |
| %TWL 12 Months | 34.4 ± 5.8 | 39.4 ± 4.9 | **0.0007** |
| %EBMIL 12 Months | 94.8 ± 19.8 | 106.1 ± 19.1 | **0.0061** |

Variables are presented in mean ± standard deviation. P25: 98.3 mL/min; P75: 114.2 mL/min.

Significant differences are highlighted in bold.

Abbreviations: eGFR, estimated glomerular filtration rate; %TWL, % total weight loss; %EBMIL, % excess BMI loss.
